# Characterising the metabolic functionality of the preterm neonatal gut microbiome prior to the onset of necrotising enterocolitis: a pilot study

**DOI:** 10.1186/s12866-024-03701-x

**Published:** 2024-12-23

**Authors:** Jonathan A. Chapman, Emily Wroot, Toby Brown, Lauren C. Beck, Nicholas D. Embleton, Janet E. Berrington, Christopher J. Stewart

**Affiliations:** 1 Translational and Clinical Research Institute, Newcastle University, 3Rd Floor Leech Building, Newcastle Upon TyneNewcastle, NE2 4HH UK; 2 Department of Pharmacology, University of Oxford, Oxford, OX1 3QT UK; 3 Newcastle Neonatal Service, Newcastle Hospitals NHS Trust, Newcastle Upon Tyne, UK; 4 Population Health Sciences Institute, Newcastle University, Newcastle Upon Tyne, UK

**Keywords:** Neonatal gut microbiome, Neonatal health, Necrotising enterocolitis, Gut microbiome development, Microbiome functionality, Microbiome metabolism

## Abstract

**Background:**

Necrotising enterocolitis (NEC) is a devastating bowel disease that primarily occurs in infants born prematurely and is associated with abnormal gut microbiome development. While gut microbiome compositions associated with NEC have been well studied, there is a lack of experimental work investigating microbiota functions and their associations with disease onset. The aim of this pilot study was to characterise the metabolic functionality of the preterm gut microbiome prior to the onset of NEC compared with healthy controls.

**Results:**

Eight NEC infants were selected of median gestation 26.5 weeks and median day of life (DOL) of NEC onset 20, with one sample used per infant, collected within one to eight days (median four) before NEC onset. Each NEC case was matched to a control infant based on gestation and sample DOL, the main driver of microbiome composition in this population, giving a total cohort of 16 infants for this study. Dietary exposures were well matched. The microbiota of NEC and control infants showed similar wide-ranging metabolic functionalities. All 94 carbon sources were utilised to varying extents but NEC and control samples clustered separately by supervised ordination based on carbon source utilisation profiles. For a subset of eight samples (four NEC, four control) for which pre-existing metagenome data was available, microbiome composition was found to correlate significantly with metabolic activity measured on Biolog plates (*p* = 0.035). Comparisons across all 16 samples showed the NEC microbiota to have greater utilisation of carbon sources that are the products of proteolytic fermentation, specifically amino acids. In pairwise comparisons, L-methionine was highly utilised in NEC samples, but poorly utilised in controls (*p* = 0.043). Carbon sources identified as discriminatory for NEC also showed a greater enrichment for established markers of inflammatory disease, such as inflammatory bowel disease, irritable bowel syndrome and diverticular disease.

**Conclusions:**

Before NEC onset, the preterm gut microbiota showed greater metabolic utilisation of amino acids, potentially indicating a shift from predominantly saccharolytic to proteolytic fermentation. Products of amino acid breakdown could therefore act as biomarkers for NEC development. A larger study is warranted, ideally with infants from multiple sites.

**Supplementary Information:**

The online version contains supplementary material available at 10.1186/s12866-024-03701-x.

## Background

Necrotising enterocolitis (NEC) is a serious bowel disease occurring in 5–10% of infants born < 32 weeks gestation and is a leading cause of preterm mortality [1–3]. Clinical presentation and course vary from patient to patient, making early diagnosis and intervention difficult. Additionally, the biological mechanisms that lead to NEC development are imperfectly understood [4]. However, abnormal development of the gut microbiome has been implicated, although no individual causative agent has been identified [5–14]. Neonates diagnosed with NEC tend to have reduced microbial diversity within their gut microbiomes, which includes a reduction in members of the phylum Actinobacteria, such as *Bifidobacterium*, and an increase in the phylum Proteobacteria, such as *Enterobacter* and *Klebsiella*.

This extensive characterisation of the NEC-associated microbiome has been enabled by advances in sequencing technologies. Microbial genes identified by metagenomic sequencing provide information on the metabolic functional potential (i.e., genetic capacity) of bacteria, but do not identify which genes are actually expressed nor prove metabolic activity. Previous studies have sought to address this by using proteomics to identify microbial phenotypic markers associated with NEC [12, 15]. An alternative approach of investigating substrates the NEC-associated microbiome has the metabolic capacity to utilise could also provide clinically relevant information.

**Fig. 1:**
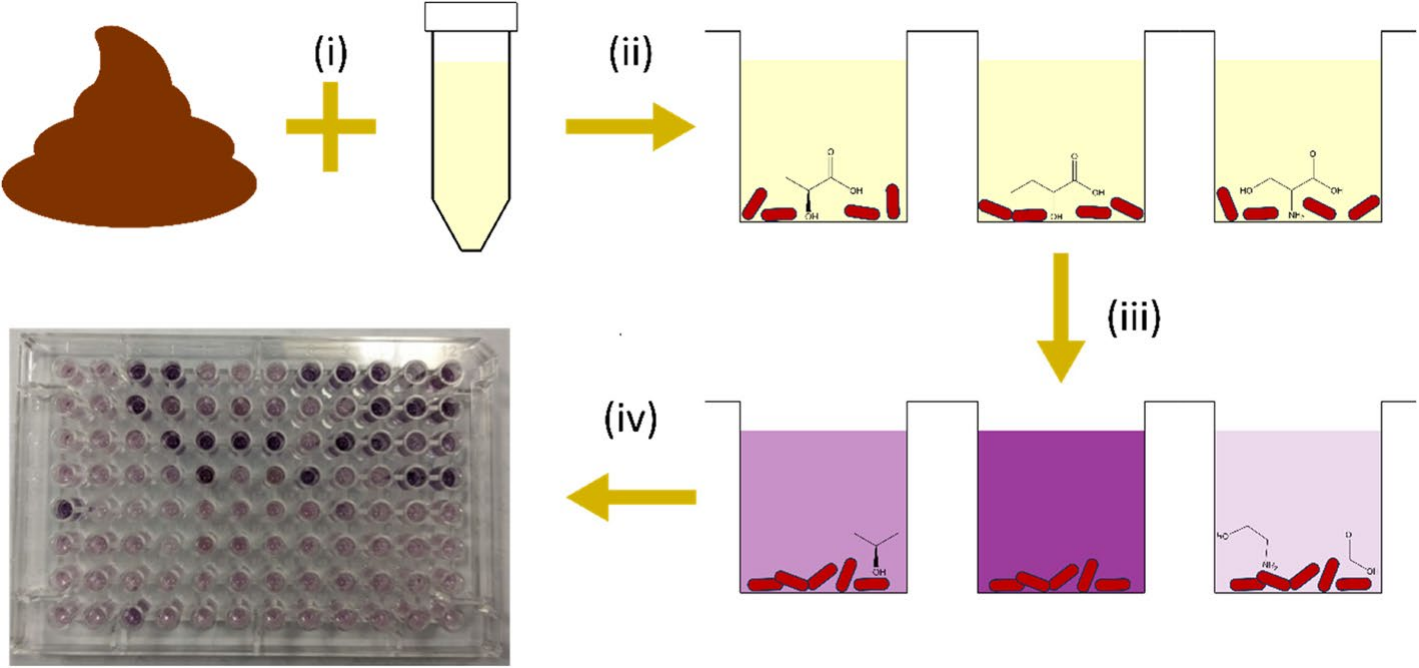
Method for assessing carbon source utilisation by neonatal gut microbiome communities. (i) Neonatal stool was suspended in anaerobic Biolog Inoculation Fluid. (ii) Stool suspension was added to each well of a Biolog AN Microplate containing a different carbon source (iii) The plate was incubated in a plate reader, within an anaerobic chamber, at 37°C for 24 h. Redox dye in the plate changes colour, giving a measure of bacterial metabolic activity and thus carbon source utilisation. (iv) After incubation, dye change in each well gives a plate-wide pattern of carbon source utilisation for the microbial community

We compared the metabolic function of the microbiota of infants who developed NEC to gestational age (GA) and day of life (DOL) matched non-NEC controls from the same unit. Biolog AN Microplates (Biolog, Inc., CA, USA) were selected as the assay method for characterisation of microbiota metabolism (Fig. 1), due to their relative ease of use, wide range of available carbon sources and applicability to higher throughput. In the published literature, this assay system has been applied in other contexts to study the metabolism of complex bacterial communities, for instance in dental plaques, but to our knowledge has not yet been used to study the neonatal microbiome [16–19].

Here we present a pilot study in which for the first time we directly investigated the metabolic functionality of the NEC gut microbiome. More broadly, we evaluated the use of the Biolog AN Microplate as an assay kit for microbiome function in stool samples.

## Methods

### Infants and sample selection

Infants contributing samples were all participating in the Research Ethics Committee approved SERVIS (NRES Committee North East and North Tyneside 2 10/H0908/39) after signed parental consent. NEC diagnoses were made using an extensive combination of clinical, X-ray and histological findings and blindly agreed by two neonatal clinicians [20]. NEC was confirmed through histology for infants who underwent surgery. One stool sample was selected per NEC case, between one and eight days (median: 4; IQR: 2–6.25) before NEC onset. Control samples were selected from GA and DOL matched infants. For patients who received probiotics, the product administered for all was Labinic (Biofloratech Ltd). Sample collection involved stool being taken from the nappy and immediately stored at -20°C, then moved to -80°C soon after.

### AN microplate assay with stool samples

AN Microplates (Biolog, Inc.) consist of a range of carbon sources, each dried into a separate well on a 96-well plate. An inoculum is then mixed with Inoculation Fluid (IF) (Biolog, Inc.) and added to each well. The wells of the plate contain a redox dye that changes colour as electrons flow through bacterial electron transport chains, thus allowing metabolic activity to be indirectly quantified by measuring absorbance with a plate reader. A microplate reader (Cerillo) was placed into an anaerobic chamber (Coy Lab Products) and left to equilibrate to 37°C for at least one hour. Following the guidance from the manufacturer, the AN Microplate was first unsealed from its packaging and exposed to the air for 30 min. It was then moved into the anaerobic chamber, along with three tubes of anaerobic IF. The IF aliquots were pooled into a single 50 mL centrifuge tube. 75 mg of patient stool sample was weighed out into a sterile microfuge tube and transferred into the 50 mL tube containing the IF in the anaerobic chamber. The stool was resuspended in the IF by vigorous pipetting and vortexing with 10 s pulses until a visually even suspension was created. The suspension was then passed through a 40 μm nylon sieve (Corning) into a new 50 mL tube to remove non-soluble pieces of stool that could impact plate reader measurements downstream. The solution was then poured into a plastic pipetting reservoir and 100 μL added to each well of the AN Microplate, using a multichannel pipette. The plate was then sealed with a Breathe-Easy gas permeable membrane (Diversified BioTech) and placed into the plate reader and incubated with no shaking for 24 h. Readings were taken at 600 nm, every 3 min. This procedure was repeated with two AN Microplates, using the remaining volume of stool suspension, so all biological repeats were run on the same day.

### Processing of raw plate reader data

Raw data were imported into R (version 4.0.4) for analysis. For each carbon source, the area under the curve (AUC) for time vs. absorbance was calculated, using a trapezoidal model, calculated with the trapz() function from the caTools package (version 1.18.2). Microbial utilisation of unknown carbon sources present in the stool samples was accounted for by subtracting the AUC calculated for the “no carbon source” blank well from the AUCs of the carbon sources. Thus, any AUC measured above that calculated for the blank well should be attributable to additional metabolic activity generated by the carbon source present in a well. After blank correction, the average well AUC (AWAUC) was calculated for the plate by summing the blank corrected AUCs and dividing by the number of wells in the plate. We often observed a spike in absorbance that went beyond the detection limit of the plate reader for the well containing Glycyl-L-Proline. This was generally seen in only one of the three repeats for a sample. Due to this inconsistent absorbance spike, seen across multiple samples, it was decided to exclude the Glycyl-L-Proline well from all downstream analyses. Accordingly, AWAUC for each plate was calculated by dividing by 95 wells, instead of the full 96. Blank corrected AUCs were then divided by the AWAUC, to give normalised AUCs. This normalisation approach allowed comparison of AUCs between plates, as different absolute numbers of bacterial cells would have been loaded, which will have impacted the metabolic activity measured. MetaboAnalyst 5.0 [21] was then used to perform principal component analysis (PCA) and dendrogram analyses on all replicates, to identify outliers. Once outliers were removed, the mean AUCs for each sample were calculated from the remaining replicates.

### Data analysis

PCA, dendrogram, orthogonal partial least squares-discriminant analysis (OPLSDA) variable influence on projection (VIP) score and metabolite enrichment analyses on the mean AUC data for all samples were performed using MetaboAnalyst 5.0, using the “statistical analysis [one factor]” option. Generalised Procrustes Analysis (GPA) was performed on metagenome abundance and Biolog datasets in R, using the package vegan (version 2.6.4). For analyses performed using MetaboAnalyst, plots were downloaded directly. Other plots were created using the ggplot2 package in R.

### Statistics

*P* values were calculated for group comparisons for DOL, GA and birthweight using logistic regression, while Pearson’s Chi Squared test was used for sex, delivery mode, probiotic receipt, antibiotics receipt and milk exposures. Pairwise comparisons between NEC and controls for mean AUCs of individual carbon sources were performed using an unpaired Student’s T test in R, with *p* < 0.05 set at the threshold for statistical significance. P values were adjusted for multiple comparisons using the false discovery rate (FDR) method, with a significance threshold of < 0.1. The same was also done for pairwise comparisons of mean AUCs for specific chemical groups. For GPA of metagenome and Biolog datasets, pairwise comparisons were performed using the protest() function of the vegan package, with 10000 permutations.

## Results

### Metabolic activity measured on biolog plates correlates with microbiome composition

**Fig. 2:**
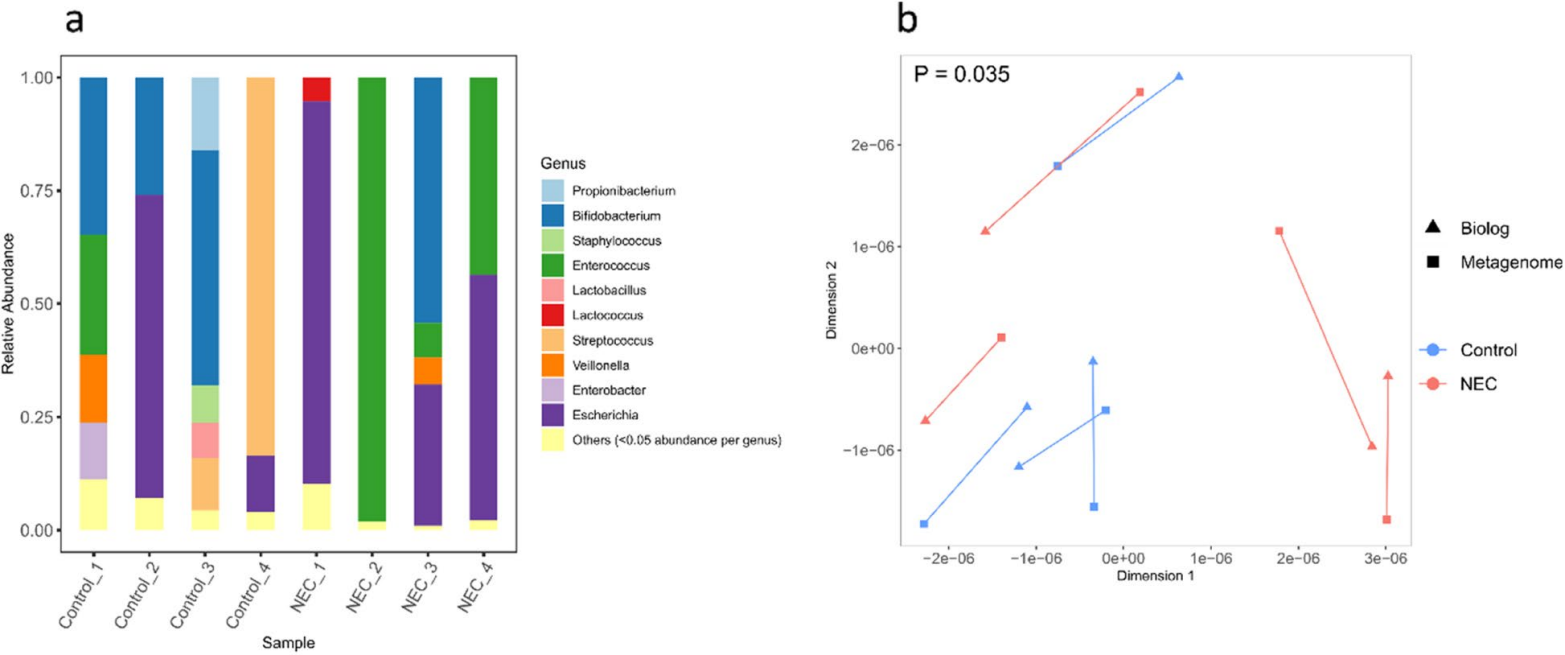
Comparison of Biolog functional activity with microbiome profiles from the same stool samples. Analysis includes a subset of eight samples (four NEC and four controls) for which metagenome data was available. **a** Stacked bar plots of microbiota composition at the genus level. Samples names with the same number are matched NEC-Control pairs. Genera with a relative abundance < 0.05 are pooled into the “Others” category. **b** Ordination plot showing outcome of Generalised Procrustes Analysis (GPA) comparing taxonomic data at the genus level with data generated from Biolog assays for the same subset of eight samples as in (**a**)

For a subset of eight samples (four NEC and four control), genus-level relative abundance data was already available (Fig. 2a), from metagenomic sequencing performed as part of another study [22]. These data were used to validate the ability of Biolog assays to measure unique metabolic utilisation patterns for individual microbiomes. GPA and subsequent ordination (Fig. 2b) of the genus-level relative abundances and the Biolog data for the eight samples showed a significant correlation between the two datasets (Procrustes sum of squares = 0.49; correlation in a symmetric Procrustes rotation = 0.71; *p* = 0.035).

### The preterm microbiota has broad functional metabolic activity that is different between NEC and controls

NEC and control pairs were from well matched infants, including for milk/feed exposures (Table 1). The microbiome from four (all NEC) of the sixteen samples was able to utilise all 94 carbon sources, while the remaining twelve showed no metabolic activity on up to 11 of the carbon sources (Supplementary Table 1). Based on presence/absence, there were no carbon sources uniquely utilised by all NEC and no control microbial communities, and vice versa.

**Table 1:** Demographic data

	NEC	Control	*P* value
Number of patients	8	8	─
DOL	14.5 (7–29.5)	14.5 (7–30.25)	0.97
GA (weeks)	26.5 (25–28.25)	26 (25–28.25)	0.61
Birthweight (g)	805 (587.5–957.5)	730 (665–1017.5)	0.7
Female	4 (50%)	5 (62.5%)	0.74
DOL of NEC onset	20 (11.75–36)	─	─
Days between sample and NEC onset	4 (2–6.25)	─	─
Surgical NEC	4 (50%)	─	─
Antibiotics within 7 days prior to sample	5 (62.5%)	4 (50%)	0.74
Vaginal delivery	4 (50%)	3 (37.5%)	0.7
Probiotics before sample	5 (62.5%)	5 (62.5%)	1
EBM only before sample	5 (62.5%)	4 (50%)	0.74
Formula only before sample	0	1 (12.5%)	0.32
EBM + formula before sample	1 (12.5%)	0	0.32
EBM + BMF before sample	1 (12.5%)	3 (37.5%)	0.32
EBM + formula + BMF before sample	1 (12.5%)	0 (0%)	0.32
Full feeds reached before sample	4 (50%)	4 (50%)	1

Median (IQR) is for DOL, GA, birthweight, ‘DOL of NEC onset’ and ‘Days between sample and NEC onset’; all other data is n (%)

*DOL* Day of Life, *GA* Gestational Age, *EBM* expressed breast milk, *BMF* breast milk fortifier

**Fig. 3:**
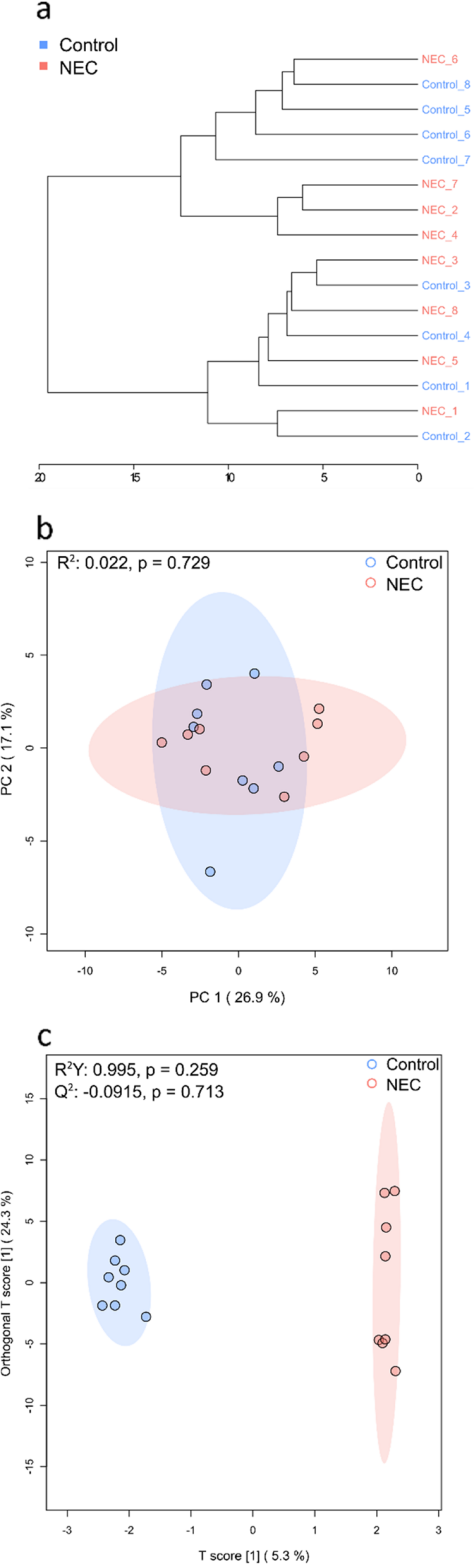
Statistical analyses of overall profiles of carbon source utilisation for each sample. **a** Dendrogram showing hierarchical clustering of the mean data for all samples, based on Euclidean distance. Sample names with the same number are matched NEC-Control pairs. **b** Principal component analysis (PCA) plot of all samples, with 95% confidence regions show. **c** Orthogonal partial least squares-discriminant analysis (OPLSDA) plot of all samples

Analysis of the overall profiles of carbon source utilisation was performed using PCA and hierarchical clustering based on Euclidean distance (Fig. 3a and b, respectively). Under these unsupervised ordination methods, samples did not cluster based on disease state. Supervised ordination was employed to uncover more subtle associations, finding NEC and control samples clustered distinctly in OPLSDA ordination based on microbial carbon source utilisation (Fig. 3c). The R^2^Y value for the model was 0.995, showing a high goodness of fit, but the corresponding Q^2^ value was -0.0915, showing poor prediction.

### Discriminatory carbon sources for NEC and controls

**Fig. 4:**
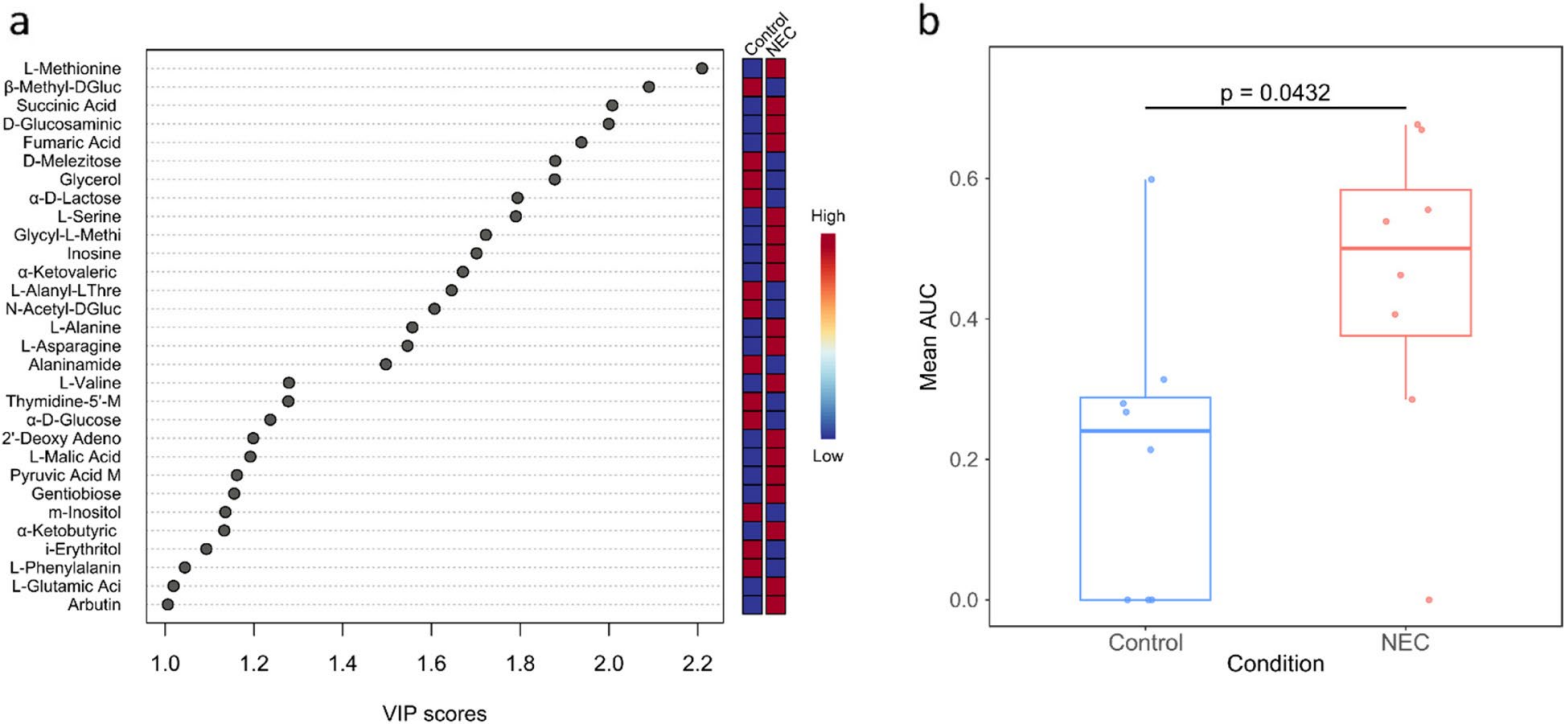
Overview of carbon sources that were most discriminatory for control or NEC. **a** Plot of variable influence on projection (VIP) scores for carbon sources contributing to discrimination between NEC and control samples in the orthogonal partial least squares-discriminant analysis (OPLSDA) ordination. **b** Box plot of per sample mean area under the curve (AUC) for metabolic activity when growing on L-methionine

A list of carbon sources that were most discriminatory between NEC and control were extracted from the OPLSDA based on the corresponding VIP scores, which measure the importance of a variable for discriminating between the two groups. In total, 30 carbon sources had a VIP score of ≥ 1, with L-methionine showing the highest VIP score (Fig. 4a). This corresponded with pairwise comparisons between NEC and controls of mean AUCs of individual carbon sources, in which L-methionine was the only statistically significant carbon source. Specifically, L-methionine was more highly utilised in NEC samples (mean AUC = 0.45) compared to controls (mean AUC = 0.21) (Fig. 4b).

### NEC microbiota showed greater utilisation of proteolytic products and a greater enrichment for biomarkers of inflammatory diseases

**Fig. 5:**
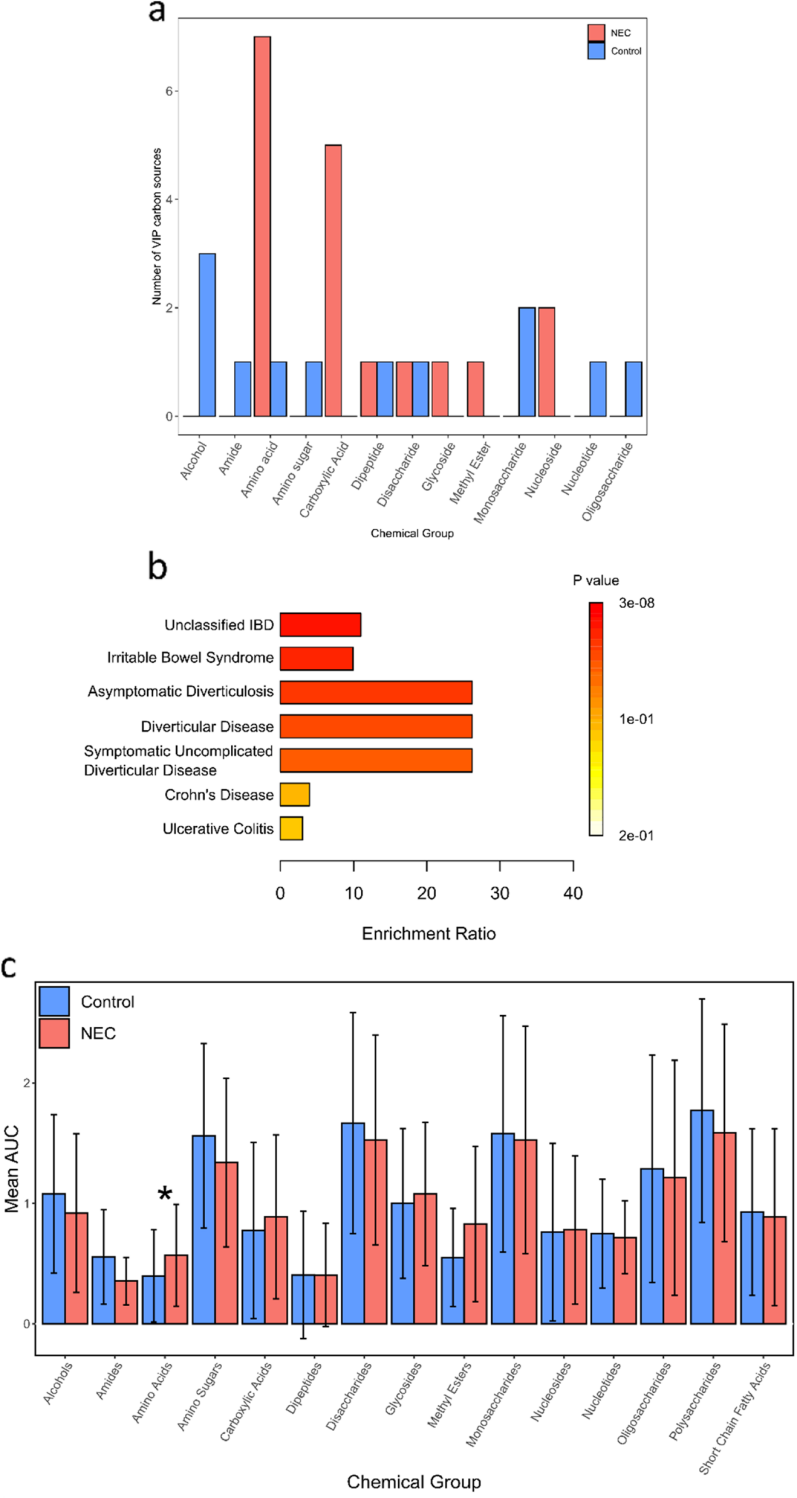
Overview of utilisation of different chemical groups across NEC and control, and NEC carbon source enrichment analysis. **a** The 30 carbon sources that had variable influence on projection (VIP) scores ≥ 1, as shown in Fig. 4a, categorised based on chemical group and subdivided based on whether they were highly utilised in NEC or control samples. **b** Enrichment ratios for known stool associated markers of specific diseases within carbon sources highly utilised in NEC. **c** Comparison between NEC and control samples of the mean area under the curve (AUC) for all chemical groups present on the AN Microplate, encompassing all 94 carbon sources. * denotes comparison that passed our exploratory threshold of FDR *p* < 0.1 for statistical significance

The list of 30 carbon sources with VIP ≥ 1 was divided into those highly utilised in control (12) or NEC (18). The carbon sources highly utilised in the control group were dominated by carbohydrates (7/12), more than half of which were sugars (Fig. 5a).

They were comprised of the following chemical groups: monosaccharide (2), disaccharide (1), oligosaccharide (1) and alcohols (3). In contrast, the carbon sources highly utilised in NEC samples showed a balance between two distinct groups: proteolysis-derived compounds and carbohydrates (Fig. 5a). Of the proteolysis-derived compounds (8/18), the majority were amino acids (7), alongside a dipeptide (1). In contrast, within the highly utilised control carbon sources, only 2/12 were proteolysis-derived compounds, consisting of an amino acid and a dipeptide. Carbohydrate carbon sources highly utilised in NEC samples (8/18) were, in contrast to controls, dominated by carboxylic acids (5) rather than sugars.

The 18 VIP carbon sources that were highly utilised in NEC were cross-checked against the Metaboanalyst [21] database of known disease markers identified in human stool. This analysis showed the carbon sources that discriminated NEC from controls were enriched for markers of (adult) gastrointestinal inflammatory diseases (Fig. 5b). The most highly enriched categories were jointly ‘Diverticular Disease’, ‘Symptomatic Uncomplicated Diverticular Disease’ and ‘Asymptomatic Diverticulosis’, comprising a total of five markers, namely fumaric acid, methionine, alanine, valine and glutamic acid.

Finally, we performed global comparisons of carbon utilisation from mean AUC for all chemical groups present on the AN microplate (i.e., all 94 carbon sources) (Fig. 5c). Of the 15 chemical groups, only amino acids passed the statistical threshold for biological difference between NEC and control (*p* = 0.005, FDR *p* = 0.08). The average utilisation of an amino acid in NEC was 42.5% higher compared to control (mean amino acid AUC = 0.57 vs. 0.40, respectively). This corresponds with the higher prevalence of amino acids amongst the VIP carbon sources that were highly utilised in samples collected before NEC diagnosis.

## Discussion

We aimed to investigate differences in metabolic function of the preterm gut microbiome between NEC and healthy infants. We characterised the metabolic functionality of gut microbial communities present in stool samples using Biolog AN Microplates and conducted a pilot study using eight NEC and eight non-NEC preterm infant samples. Our findings show congruence between observed metabolic functionality and microbiome composition, validating the physiological relevance of this assay method. Secondly, our results suggest that the microbial communities in infants who develop NEC have a greater utilisation of proteolytic products, in the form of amino acids, compared with healthy controls. L-methionine was the most significant discriminator between NEC and controls, with higher utilisation in NEC infants. Furthermore, the group of carbon sources that were highly utilised in NEC samples and poorly in controls were more enriched for known markers of other gastrointestinal diseases.

A study of 50 preterm neonates previously observed divergence of their microbiomes into distinct saccharolytic and proteolytic fermentation models based on initial feeding method [23]. In the current study, NEC and healthy cohorts were well matched for overall feed exposures (Table 1). Our results therefore suggest that the potential shift to proteolytic fermentation by the NEC microbiome is at least partially mediated by other factors.

A shift in the microbiota from predominantly saccharolytic to proteolytic fermentation prior to the onset of NEC, as implied by the greater metabolic utilisation of amino acids in NEC samples, points to a set of potential biomarkers that could be used to detect disease onset. A previous study found five amino acids, including methionine, to be higher in preclinical faecal samples of infants who developed NEC, suggesting that there may indeed be a greater abundance of these metabolites present in the neonatal gut prior to NEC onset for members of the gut microbiota to ferment [24]. The link between elevated methionine levels and NEC onset is intriguing, as methionine can be metabolized into bacterial quorum-sensing molecules, and dietary restriction of this amino acid has been proposed to be protective against enteric infection in animal models through disrupting pathogenic quorum signalling [25].

More broadly, catabolism of amino acids by microbes in the gut produces ammonia, branched chain fatty acids (BCFAs), sulfides, phenols, indoles and amines [26–29]. Monitoring levels of such compounds in stool could serve as an additional diagnostic tool to aid the early detection of NEC. This is supported by a study that performed proteomic profiling of neonatal stool samples to predict NEC, in which numerous proteolytic proteins were more highly abundant before NEC onset [15]. The data presented supports and expands this previous work through identifying a potential increase in utilisation of proteolytic products by gut microbes prior to NEC onset.

With the exception of BCFAs, the products of amino acid breakdown are pro-inflammatory and cytotoxic, as highlighted in extensive reviews elsewhere [30–33]. Catabolism of L-methionine, highly utilised in NEC samples and the only carbon source to pass our statistical threshold, serves as an illustrative example. This process produces methanethiol and hydrogen sulfide, which cause cytotoxicity and have been associated with IBD and perturbation of the gut microbiota [31, 34]. Amino acid catabolism by gut microbes could therefore be contributing to the pathology of NEC, by promoting inflammation and cell death within the intestinal epithelium.

This study has several important limitations. The sample size used, while appropriate for a pilot study, is small. These findings require further validation as the neonatal microbiome is known to be most heavily influenced by the Neonatal Intensive Care Unit (NICU) in which the baby is cared for, meaning that findings may differ between NICUs [35]. Furthermore, the supervised analytical methods used here are more susceptible to overfitting when sample sizes are small, hence the high R^2^Y but low Q^2^ values for our model. A potential limitation is the stool samples were frozen after collection, which may have impacted the viability of some microbes and the subsequent metabolic function of the microbiome. Validation on fresh stools would be ideal, but virtually impossible as the point at which NEC may or will develop is unknown. It should also be noted that NEC onset can be associated with viral infections, and in focusing on bacterial members of the gut microbiome, this study does not address potential viral mechanistic triggers of the disease [36]. The use of the Biolog Microplate assay in isolation also presents limitations in comparison to proteomic approaches, as it cannot identify the effectors produced by bacteria that are responsible for observed differences in carbon source utilisation. Finally, the assay plates were only incubated for 24 h, meaning the metabolic activity measured may weakly reflect the contribution of the metabolism of obligate anaerobes.

## Conclusions

In summary, we used stool samples to trial Biolog AN Microplates in a novel application for the study of changes in metabolic functionality of the preterm gut microbiome prior to the onset of NEC. Our results indicate a potential shift towards proteolytic fermentation and amino acid catabolism prior to NEC diagnosis. This may offer novel biomarkers for the early diagnosis of NEC in the form of amino acid breakdown products, but requires validation in a larger sample size and infants from different units. Furthermore, production of amino acid catabolites in the neonatal gut should be investigated further, as they may contribute to NEC pathology.

## Supplementary Information

Supplementary Material 1.

## Data Availability

Data for each sample from the Biolog AN Microplates, upon which all analyses were based, are available within the supplementary information file.
